# Combined analysis of gut microbiome and serum metabolomics reveals novel biomarkers in patients with early-stage non-small cell lung cancer

**DOI:** 10.3389/fcimb.2023.1091825

**Published:** 2023-01-20

**Authors:** Boxiong Ni, Xianglong Kong, Yubo Yan, Bicheng Fu, Fucheng Zhou, Shidong Xu

**Affiliations:** Department of Thoracic Surgery, The Third Affiliated Hospital of Harbin Medical University, Harbin, China

**Keywords:** early-stage NSCLC, IPA, gut microbiome, serum metabolite, biomarkers

## Abstract

Non-small cell lung cancer (NSCLC) is the predominant form of lung cancer and is one of the most fatal cancers worldwide. Recently, the International Association for the Study of Lung Cancer (IASLC) proposed a novel grading system based on the predominant and high-grade histological patterns for invasive pulmonary adenocarcinoma (IPA). To improve outcomes for NSCLC patients, we combined serum metabolomics and fecal microbiology to screen biomarkers in patients with early-stage NSCLC and identified characteristic microbial profiles in patients with different grades of IPA. 26 genera and 123 metabolites were significantly altered in the early-stage NSCLC patients. *Agathobacter*, *Blautia*, *Clostridium*, and *Muribaculacea* were more abundant in the early-stage NSCLC patients compared with healthy controls. For the different grades of IPA, the characteristic microorganisms are as follows: *Blautia* and *Marinobacter* in IPA grade type 1; *Dorea* in IPA grade type 2; and *Agathobacter* in IPA grade type 3. In the metabolome results, the early-stage NSCLC group mainly included higher levels of sphingolipids (D-erythro-sphingosine 1-phosphate, palmitoyl sphingomyelin), fatty acyl (Avocadyne 1-acetate, 12(S)-HETE, 20-Carboxy-Leukotriene B4, Thromboxane B3, 6-Keto-prostaglandin f1alpha, Sebacic acid, Tetradecanedioic acid) and glycerophospholipids (LPC 20:2, LPC 18:0, LPC 18:4, LPE 20:2, LPC 20:1, LPC 16:1, LPC 20:0, LPA 18:2, LPC 17:1, LPC 17:2, LPC 19:0). Dysregulation of pathways, such as sphingolipid metabolism and sphingolipid signaling pathway may become an emerging therapeutic strategy for early-NSCLC. Correlation analysis showed that gut microbiota and serum metabolic profiles were closely related, while *Muribaculacea* and *Clostridium* were the core genera. These findings provide new biomarkers for the diagnosis of early-stage NSCLC and the precise grading assessment of prognostic-related IPAs, which are of clinical importance and warrant further investigation of the underlying molecular mechanisms.

## Introduction

Lung cancer, one of the deadliest malignancies, poses a huge threat to human health with increasing morbidity and mortality worldwide (Siegel et al., 2018). It consists of non-small cell lung cancer (NSCLC) and small cell lung cancer (SCLC), with NSCLC being the most common form, accounting for more than 80% of lung cancers (Herbst et al., 2018; Duma et al., 2019). Lung adenocarcinoma (LADC), the most common pathologic type of NSCLC, is an important factor that discriminates patient prognosis (Travis et al., 2015). Recently, a novel grading system based on the predominant and high-grade histological patterns for invasive pulmonary adenocarcinoma (IPA) has been proposed by the International Association for the Study of Lung Cancer (IASLC) (Moreira et al., 2020). The model has consistently been found to correlate with prognosis and consists of: Grade 1: lepidic predominant tumor; Grade 2: acinar or papillary predominant tumor, both with no or less than 20% of high-grade patterns; and Grade 3: any tumor with 20% or more of high-grade patterns (solid, micropapillary and or complex gland). The established grading system is based on prognostic-related histological criteria and has utility and prognostic significance for IPA (Hou et al., 2022). Importantly, most lung cancer patients are initially diagnosed at an advanced stage of the disease, often with a poor prognosis. Therefore, developing biomarkers with high sensitivity and specificity to assess lung cancer progression and treatment effects will greatly improve disease management and patient survival.

The gut microbiome, recognized as the second genome of humans (Qin et al., 2010), has attracted considerable attention in recent decades. It contains more than 100 times genes than the human genome and performs key roles on human health. Dysregulation of the gut microbiota has been found to be associated with many cancers (Matson et al., 2018; Santoni et al., 2018), and disruption of metabolite balance caused by altered microbiome homeostasis may promote tumorigenesis. Recent studies have shown that the occurrence and development of NSCLC are also related to the human gut microbiota, and the interactions between these microbes can affect the function of multiple pathways including metabolism, inflammation, and immunity (Zhuang et al., 2019; Zheng et al., 2020; Lu et al., 2021; Zhao et al., 2021). These studies suggest that gut microbiota signatures have the potential to diagnose and assess the development and progression of non-small cell lung cancer.

Despite extensive progress in linking the gut microbiome to lung disease (the ‘gut-lung axis’) (Keely et al., 2012; Dumas et al., 2018; Zhang et al., 2020a), so far, the interactions between the gut microbiome and metabolome in patients with early-stage NSCLC have not been reported. Here, we recruited 43 patients with early-stage non-small cell lung cancer and 35 healthy individuals, and their stool and serum samples were tested and analyzed accordingly. Comparing the composition of gut microbiota and serum metabolites by bioinformatics analysis to search for early pathogenesis and potential biomarkers in patients with non-small cell lung cancer. On the other hand, we sought to link gut microbiota changes with a novel grading system for pulmonary adenocarcinoma, thereby providing a rationale for accurate diagnosis and typing of early-stage lung cancer.

## Materials and methods

### Study design and samples

A total of 78 participants who came to the Department of Thoracic Surgery of the Third Affiliated Hospital of Harbin Medical University were recruited, including 43 patients with early-stage non-small cell lung cancer and 35 healthy relatives of these patients (**Table 1**). Sixty-three serum (35 NSCLC and 28 healthy) and seventy-eight stool (43 NSCLC and 35 healthy) samples were collected. Fecal and serum samples were collected according to protocols approved by the local ethics committee, and written informed consent was obtained from all participants.

**Table 1:** Characteristics of health people and early-stage NSCLC patients.

Characteristics	Early-stage NSCLC (n = 43)		Healthy control (n = 35)	P value
Age (mean ± SD)	58.63 ± 9.92		55.8 ± 8.44	0.178
Male/female (No.)	18/25		18/17	0.399
BMI (kg/m^2^) (mean ± SD)	24.99 ± 3.42		24.29 ± 2.82	0.323
Tumor type, n (%)				—
ADC	38 (88.37)		—	
SCC	5 (11.63)		—	
Disease stage, n (%)				—
0	1 (2.33)		—	
I	34 (79.07)		—	
II	8 (18.6)		—	
Novel IASLC grading of IPA, n (%)				—
I	7 (16.28)		—	
II	12 (27.91)		—	
III	10 (23.26)		—	
Smoking status, n (%)				0.172
Smoker	16		8	
Non-smoker	27		27	
Tumor metastasis, n (%)				—
Non-metastasis	43 (100)		—	
Metastasis	0		—	
Family history, n (%)				0.132
Yes	27		16	
No	16		19	

Collate clinical parameter information (including age, gender, body mass index (BMI), tumor stage, novel adenocarcinoma grade, smoking, family history, etc.), and exclude any unhealthy conditions by electrocardiogram and chest X-ray results. The main exclusion criteria were as follows: (1) ≤ 18 years old or > 80 years old, (2) individuals who had received antibiotics or probiotics in the past 3 months, (3) underlying diseases such as diabetes and hypertension.

### Sample collection

Fecal and serum samples were collected in the morning after an overnight fast (≥8 h). The stool samples were divided into 3 equal parts (200 mg each), placed in sterile cryovials, and immediately transported to the laboratory for storage at -80°C. Blood samples were collected in coagulation tubes. After the blood was collected, the blood was gently mixed up and down for about ten times, and then centrifuged at 1800g for 10 minutes. The supernatant (serum) was collected in a 1.5 ml centrifuge tube, centrifuged at 13,000 g for 2 min, and the supernatant was transferred to a cryovial and stored at -80°C for further analysis.

### DNA extraction

DNA from different samples was extracted using the CTAB according to manufacturer ‘s instructions. Analyze the integrity and fragment size of the extracted DNA using 1% agarose gel electrophoresis. And NanoDrop 2000 (Thermo Scientific, USA) was used to measure the extracted DNA quality.

### 16S rDNA sequencing

PCR amplification was performed using the following primers: 341F (5’-CCTACGGGNGGCWGCAG-3’) and 805R (5’- GACTACHVGGGTATCTAATCC -3’). The 5’ ends of the primers were tagged with specific barcodes per sample and sequencing universal primers. And then the PCR products were purified by AMPure XT beads (Beckman Coulter Genomics, Danvers, MA, USA) and quantified by Qubit (Invitrogen, USA). The amplicon pools were prepared for sequencing and the size and quantity of the amplicon library were assessed on Agilent 2100 Bioanalyzer (Agilent, USA) and with the Library Quantification Kit for Illumina (Kapa Biosciences, Woburn, MA, USA), respectively. The libraries were sequenced on NovaSeq PE250 platform.

### Microbiome data analysis

Paired-end reads were assigned to samples based on their unique barcodes and truncated by cutting off the barcode and primer sequence. Merge paired-end reads using FLASH (Reyon et al., 2012). Raw reads were quality filtered according to fqtrim (v0.94) under specific filter conditions to obtain high quality clean labels. Chimeric sequences were filtered using Vsearch software (v2.3.4) (Caporaso et al., 2010). After dereplication using DADA2, the representative sequence with single-base accuracy is obtained, that is, the ASV (Amplicon Sequence Variants) feature table and feature sequence. Alpha diversity and beta diversity were calculated by QIIME2 after random normalization to the same sequences, and the graphs were drawn by R package. Blast was used for sequence alignment, and the characteristic sequences of each representative sequence were annotated with the SILVA database. LEfSe (Segata et al., 2011) analysis and Wilcoxon rank sum tests were used to identify genera that were differentially abundant between groups of subjects. Other diagrams were implemented using the R package (v3.5.2) and GraphPad Prism software.

### Analysis of serum samples

Metabolites in serum samples were extracted using 80% methanol buffer. 400 µL of pre-chilled 80% methanol was added to 100 µL of the sample, vortex for 1 min, incubated for 5 min at room temperature, then overnight at -20°C. After centrifugation at 4000 g for 20 min, the supernatant was transferred to a new 96-well plate. QC samples were prepared by pooling together 10 μL of each extract. Metabolites were stored at -80°C prior to liquid chromatography-mass spectrometry (LC-MS) analysis (Want et al., 2006; Barri and Dragsted, 2013).

### Non-targeted metabolomics analysis

UHPLC-MS/MS analyses were performed using a Vanquish UHPLC system (Thermo Fisher, Germany) coupled with an Orbitrap Q ExactiveTMHF-X mass spectrometer (Thermo Fisher, Germany). The sample was injected onto a Hypesil Gold column (100 × 2.1 mm, 1.9 μm) using a 12-minute linear gradient at a flow rate of 0.2 mL/min. The eluents for positive polarity mode were 0.1% formic acid–water (A) and methanol (B). The eluents used for negative polarity mode were 5 mM pH 9.0 ammonium acetate (A) and methanol (B). The Q ExactiveTM HF-X mass spectrometer was operated in positive/negative mode with a spray voltage of 3.5 kV, a capillary temperature of 320°C, a sheath gas flow of 35 psi, an auxiliary gas flow of 10 L/min, S-lens RF class 60, Auxiliary gas heater temperature 350°C.

### Metabolomic data analysis

Statistical analysis was performed using statistical software R (R version R-3.4.3), Python (Python 2.7.6 version) and CentOS (CentOS 6.6 version). When the data were not normally distributed, area normalization was used for positive state transformation method.

These metabolites were annotated using the following databases: the KEGG database (https://www.genome.jp/kegg/pathway.html), the HMDB database (https://hmdb.ca/metabolites) and the LIPIDMaps database (http://www.lipidmaps.org/). Partial least squares discriminant analysis (PLS-DA) was performed in metaX (Wen et al., 2017). Metabolites with VIP > 1 and P value < 0.05 and fold change (FC) ≥ 1.2 or FC ≤ 0.833 were considered differential metabolites. Volcano plots were used to filter metabolites of interest based on log2 (fold change) ≥ 0.263 or log2 (fold change) ≤ -0.263, and -log10 (P-Value) metabolites from ggplot2 in R language. For cluster heatmaps, data were normalized using z-scores of regions of differential metabolite intensity and plotted in R by the heatmap package. The functions of these metabolites and metabolic pathways were investigated using the KEGG database. Metabolic pathway enrichment of differential metabolites was carried out.

### Statistical analysis

Patient characteristics were expressed as mean ± standard deviation (SD), differences between groups were compared using the χ^2^ test or independent samples *t*-test. Wilcoxon rank-sum test (for two groups) and Kruskal-Wallis test (for more than two groups) were used to compare differences among microbial groups. Student’s *t*-test and fold change analysis were used to compare metabolites between groups. The relationship between microorganisms and metabolites was assessed using Spearman rank correlation analysis. Values of P < 0.05 were considered as statistically significant.

## Results

### Gut microbial profile of early-stage NSCLC patients

To determine whether gut microbial changes were associated with early-stage NSCLC, we examined different groups of fecal microbiome samples, including 43 NSCLC patients and 35 healthy individuals, by 16S rRNA gene sequencing. All patients with non-small cell lung cancer are in the early stage and have not developed distant metastasis, including stage 0 (adenocarcinoma in situ, AIS) (2.33%), stage I (79.07%), and stage II (18.6%). The detailed clinical characteristics of all participants are shown in **Table 1**. There were no significant differences in age, gender, smoking status body mass index (BMI) and family history between the two groups (P > 0.05).

**Figure 1:**
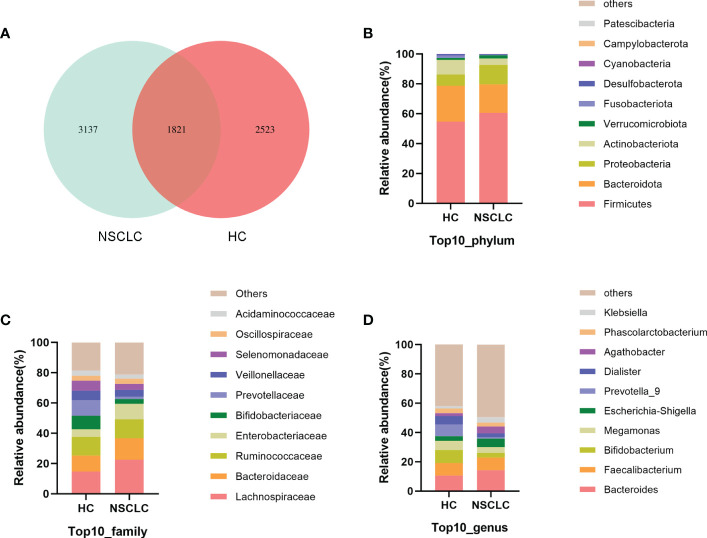
The characteristics of gut microbiota community structure **(A)** The Venn diagram shows unique and common ASVs in early-stage NSCLC and HC. **(B–D)** The top 10 representative species and their proportions in the two groups at the level of phylum **(B)**, species **(C)**, and genus **(D)**.

Using amplicon sequence variants (ASVs) to track the dynamics of bacterial abundance in feces from different groups, Venn plots visualized the number of ASVs shared and unique between the healthy control (HC) group and the early-stage NSCLC group (**Figure 1A**). We found the two groups shared 1821 ASVs, and the early-stage NSCLC group had more unique ASVs than HC group (**Supplementary Table 1**). And then we analyzed the community structure of gut microbes (**Supplementary Table 2**). At the phylum level, *Firmicutes*, *Bacteroidota*, *Proteobacteria*, and *Actinobacteriota* were the main components in both the HC group and early-stage NSCLC group, with the abundance of *Firmicutes* and *Proteobacteria* being higher in the NSCLC group (**Figure 1B**). At the family level, compared to the HC group, the abundance of *Lachnospiraceae*, *Bacteroidaceae*, and *Enterobacteriaceae* were higher in early-stage NSCLC group, while the abundance of *Bifidobacteriaceae*, *Prevotellaceae* and *Veillonellaceae* were lower (**Figure 1C**). At the genus level, apart from the similar abundance of *Faecalibacterium* in both HC group and NSCLC group, *Bacteroides* and *Escherichia-Shigella* were slightly more abundant in early-stage NSCLC group, while *Bifidobacterium*, *Megamonas*, *Prevotella_9* and *Dialister* were relatively lower (**Figure 1D**).

**Figure 2:**
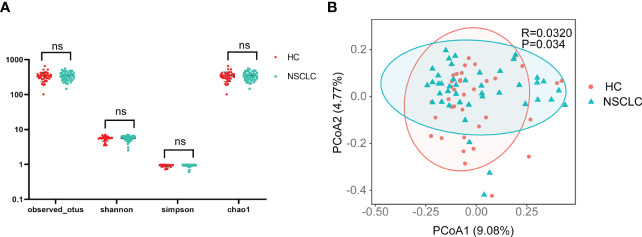
Comparison of α- and β-diversity of gut microbiota in HC and early-stage NSCLC groups **(A)** Differences in α diversity between early-stage NSCLC and HC based on the observed outs, shannon, simpson and chao1. **(B)** PCoA shows β diversity differences between the two groups (Bray-Curtis, R = 0.032, P< 0.05).

Next, statistical analysis of microbial abundance was performed. Both early-stage NSCLC group and HC group showed comparable numbers of observed OTUs (operational taxonomic units). The Shannon and Simpson indexes both showed that community diversity was similar among the two groups. The Chao1 index showed no significant differences in community richness between early-stage NSCLC group and HC group. These data suggest that global community alpha diversity is similar between early-stage NSCLC group and HC group (**Figure 2A**). When comparing microbial community structure, beta diversity showed differences between the two groups (**Figure 2B**).

### Specific gut microbiome signatures in early-stage NSCLC patients

**Figure 3:**
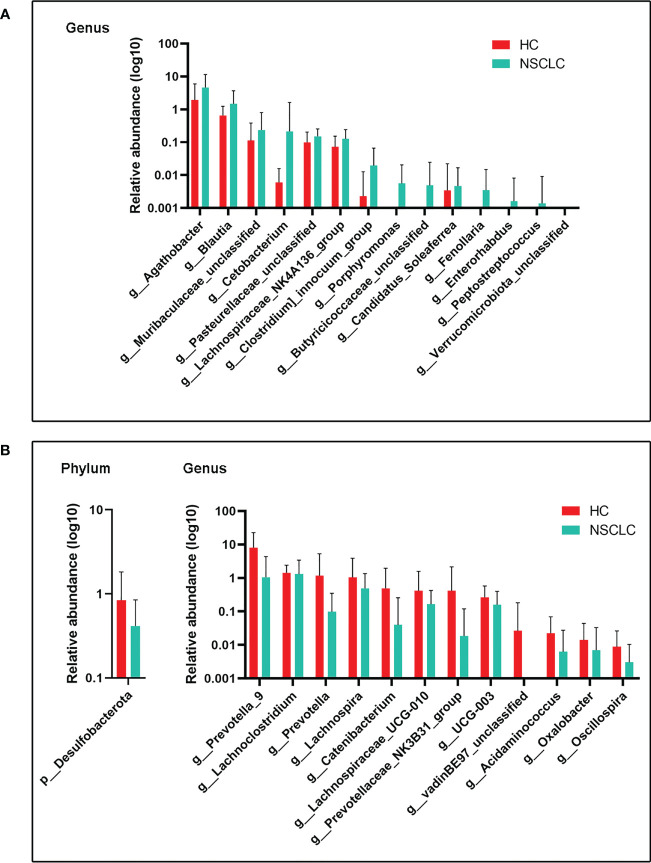
Differences in gut microbiota abundance between early-stage NSCLC and HC **(A)** Increased microbiota abundance in early-stage NSCLC at the genus level (P< 0.05). **(B)** Decreased microbiota abundance in early-stage NSCLC at the phylum and genus levels (P< 0.05). P values were calculated using the two-tailed Wilcoxon rank-sum test.

We next compared gut microbes with significant differences in expression abundance between HC and early-stage NSCLC groups at the phylum and genus levels. In total, 1 phylum and 12 genera were significantly decreased in the abundance of early-stage NSCLC patients (**Figure 3B**), while 14 genera were conversely enriched (**Figure 3A**). *Desulfobacterota*, the only phylum with significant differences between these two groups, was more abundant in the HC group. At genus level, *Agathobacter*, *Blautia*, *Clostridium*, an uncharacterized genus of family *Muribaculacea*, *Cetobacterium*, an uncharacterized genus of family *Pasteurellaceae* and eight other genera were significant abundant in early-stage NSCLC than in HC group, whereas *Lanchnoclostridium*, *Prevotella*, *Lachnospia*, *Catenibacterium*, *Oscillospira*, *UGG-003*, *Lachnospiaceae_UGG-010*, an uncharacterized genus of family *vandinBe97*, *Acidaminococcus*, *Prevotellaceae_NK3B31*_group, *Oxalobacter* were significantly enriched in the HC group.

**Figure 4:**
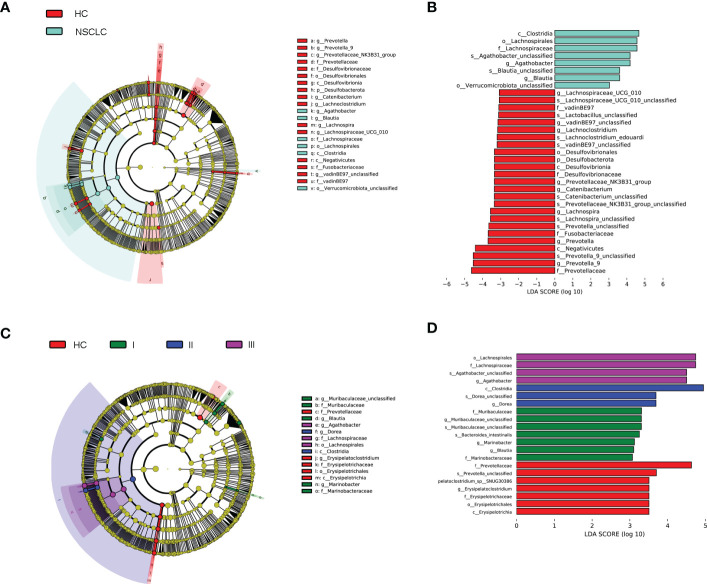
Linear discriminant analysis (LDA) combined with effect size (LEfSe). **(A)** Cladograms of the phylogenetic distribution of the microbiota with significant differences between early-stage NSCLC and HC analyzed by LefSe. **(B)** Histogram of the distribution of LDA values for LEfSe analysis of intestinal flora in the two groups (LAD score ≥ 3). **(C)** Cladograms of the phylogenetic distribution of the microbiota with significant differences across IPA grade I, grad II, grade III and HC analyzed by LefSe. **(D)** Histogram of the distribution of LDA values for LEfSe analysis of intestinal flora in four groups of samples (LAD score ≥ 3). The listed bacterial floras are significantly gathered for their respective groups (P < 0.05, Kruskal‐Wallis test).

Linear discriminant analysis (LDA) effect size (LEfSe) (Segata et al., 2011) was then used to generate cladograms to reveal differences in taxa abundance between early-stage NSCLC and HC (**Figure 4A**). There were 25 and 8 bacterial taxonomic clades that were significantly different in HC and early-stage NSCLC groups, respectively [log10 (LDA score) > 3] (**Figure 4B**). We found that *Clostridia* class was significantly higher in the early-stage NSCLC group. *Agathobacter* and *Blautia* were the prominent gene level biomarkers for early-stage NSCLC group. For healthy controls, the *Desulfovibrionia* and *Negativicutes* were the abundant class, and *Prevotella_9*, *Prevotella*, *Lachnospira* and *Catenibacterium* were the most prominent genus level biomarkers. Overall, these findings demonstrated that the early-stage NSCLC group had relatively lower microbial abundance than the HC group and was sufficient enough to distinguish healthy individuals from early-stage NSCLC patients.

### Gut microbial compositions correlate with a novel grading system of IPA

Adenocarcinoma was the main type of pathology in NSCLC (38/43, 88.37%). In this study, after ruled out adenocarcinoma *in situ* (1 case) and minimally invasive adenocarcinoma (8 cases), we classified invasive pulmonary adenocarcinoma (IPA) into three groups (grade 1, n =7; grade 2, n =12; grade 3, n =10) according to the new grading system proposed by the IASLC (Moreira et al., 2020; Deng et al., 2021). Differences in microbial composition between healthy patients and IPA patients with different grades under the new grading system were analyzed at the phylum and species levels (**Supplementary Figure 2**). Relative abundance analysis showed clear distinctions between high and low abundance taxa, and used color gradients to reflect similarities and differences in the composition of multiple samples at each taxonomic level. As shown in **Supplementary Figure 2**, according to the change of the color gradient, the differences between the four groups of samples can be seen intuitively. The data showed that the dominant flora of IPA patients in each group was different from that of healthy people, suggesting a correlation between the florae features and the histopathological process of invasive lung adenocarcinoma.

Next, we analyzed biomarkers between IPA patients with different grades and healthy controls by multi-level LEfSe (**Figure 4C**). There were significant differences in 7, 7, 3, and 4 bacterial taxonomic clades in the healthy group and in the invasive pulmonary adenocarcinoma grades type 1, type 2, and type 3 group, respectively [log10 (LDA score) > 3] (**Figure 4D**). The key species were *Erysipelatoclostridium* in HC; *Blautia* and *Marinobacter* in IPA grade type 1; *Dorea* in IPA grade type 2; and *Agathobacter* in IPA grade type 3. These results showed that the fecal gut microbiota was specific for a novel graded type of invasive lung adenocarcinoma.

### General overview of the serum metabolome

Previous studies have revealed that gut microbiota has a significant impact on blood metabolite profiles (Wikoff et al., 2009; Wilmanski et al., 2019). To further explore changes in gut microbe-host interactions, we performed LC-MS/MS-based non-targeted metabolomic analysis of serum from healthy individuals and patients with early-stage NSCLC. A total of 866 metabolites were identified and quantified, including 553 positive ions and 313 negative ions (**Supplementary Table 3**).

### Differentially abundant metabolites between HC and early-stage NSCLC groups

**Figure 5:**
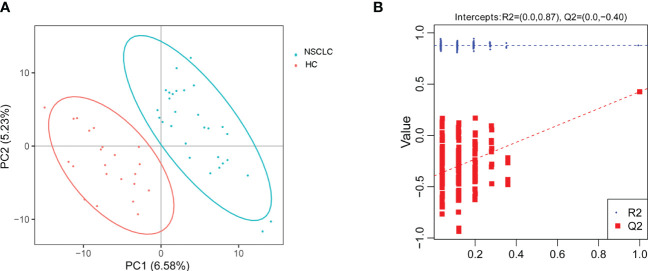
Principal component analysis. **(A)** PLS-DA score plot shows the difference in metabolites between groups. **(B)** Comparison of real and permuted model parameters in validation tests.

**Figure 6:**
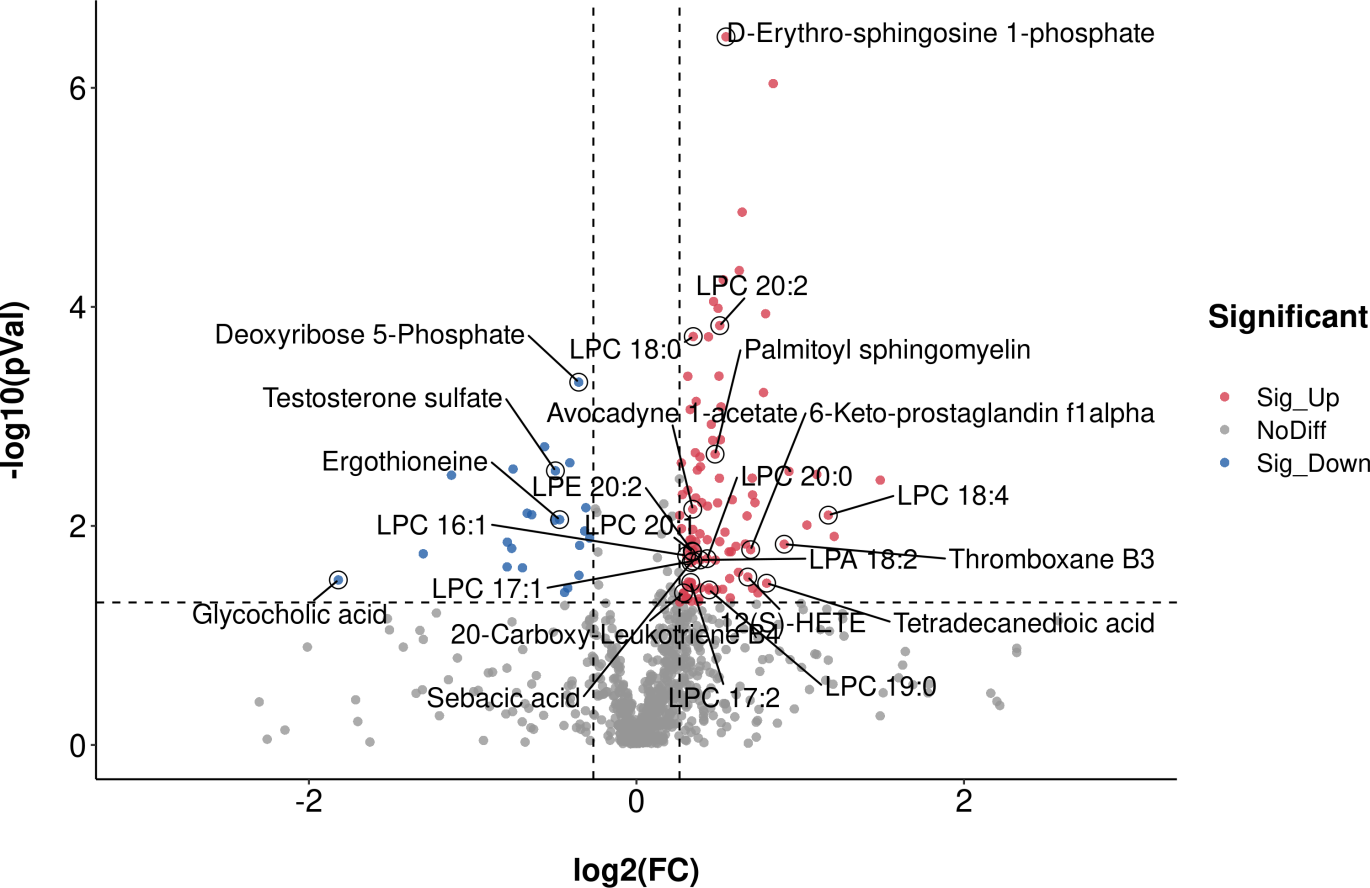
The significant different metabolites between early-stage NSCLC and HC group by Volcano plot Blue dots on the left are metabolites down-regulated in NSCLC vs HC, red dots on the right represent metabolites up-regulated in NSCLC vs HC, grey dots are metabolites that are not significantly different.

Supervised multivariate statistical analysis using partial least squares discriminant analysis (PLS-DA) to maximize screening for differential metabolites across groups. The PLS-DA score plot showed a clear separation between the HC group and the early-stage NSCLC group (**Figure 5A**). Permutation tests indicated that the data were not overfit, the R2Y and Q2 values were 0.87 and -0.4, respectively, validating the OPLS-DA model (**Figure 5B**). The differentially expressed metabolic ions are screened by p value of the t-test and variable difference contribution (VIP), where VIP≥1.0, P< 0.05 as the filter condition. A total of 123 different metabolites were identified in serum between the HC and early-stage NSCLC groups (**Supplementary Table 4**), most of which were upregulated. **Figures 6** revealed the changes in these metabolites. In the HC group, the abundant metabolites mainly included carbohydrates and carbohydrate conjugates (Deoxyribose 5-phosphate), steroids and steroid derivatives (Testosterone sulfate, Glycocholic acid, D-erythrose 4-phosphate) and amino acids, peptides and analogs (Ergothioneine). In contrast, metabolites with higher levels in the early-stage NSCLC group mainly included sphingolipids (D-erythro-sphingosine 1-phosphate, palmitoyl sphingomyelin), fatty acyl (Avocadyne 1-acetate, 12(S)-HETE, 20-Carboxy-Leukotriene B4, Thromboxane B3, 6-Keto-prostaglandin f1alpha, Sebacic acid, Tetradecanedioic acid) and glycerophospholipids (LPC 20:2, LPC 18:0, LPC 18:4, LPE 20:2, LPC 20:1, LPC 16:1, LPC 20:0, LPA 18:2, LPC 17:1, LPC 17:2, LPC 19:0). In order to show the relationship between samples and the expression differences of metabolites in the two groups more intuitively, we performed hierarchical clustering analysis, and the results for the top 50 metabolites of p values with significant differential expression were shown in **Supplementary Figure 3**.

**Figure 7:**
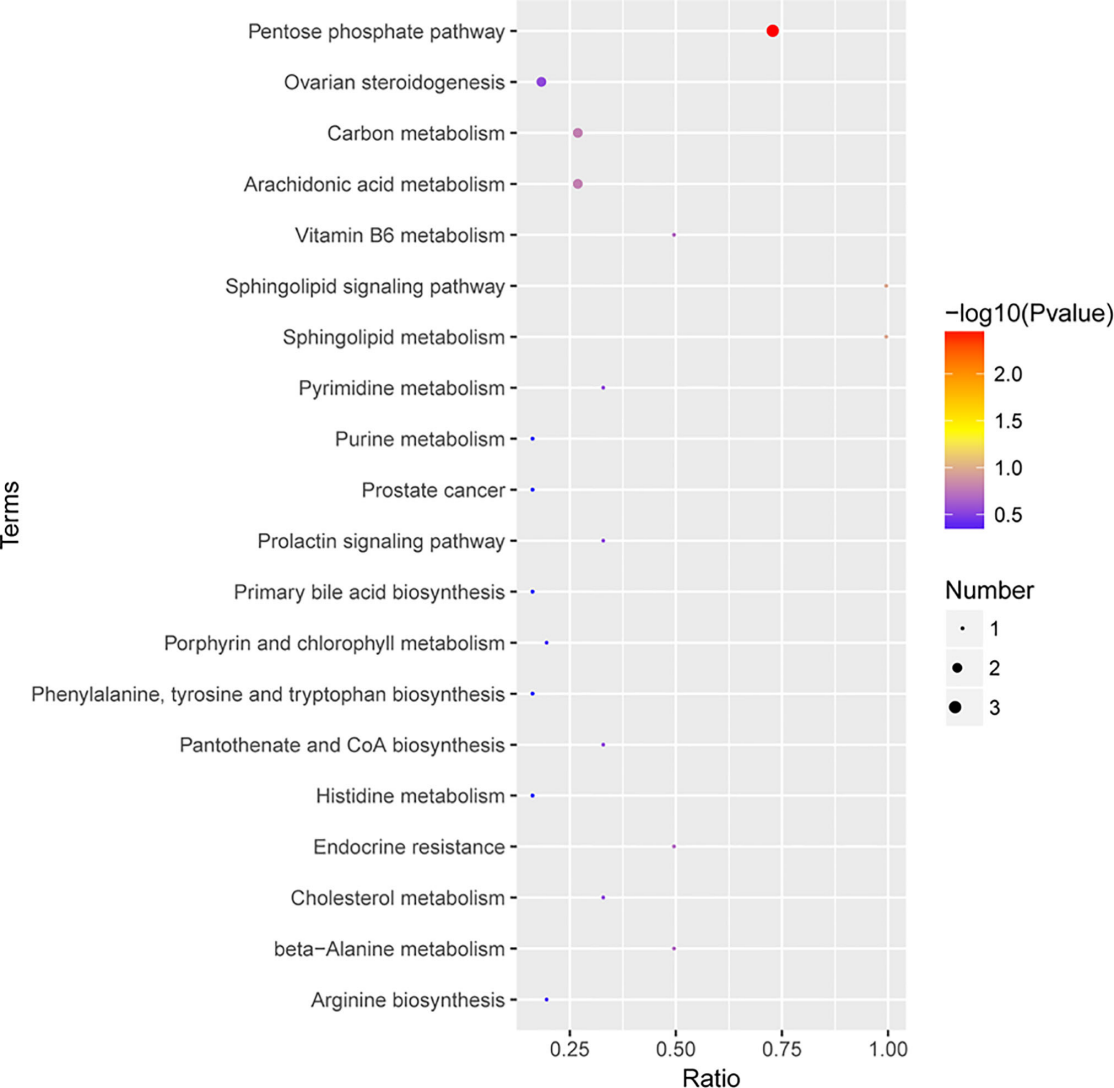
The KEGG pathway enrichment scatter plot displays important discriminatory metabolic processes of early-stage NSCLC vs HC patients.

The KEGG pathway enrichment analysis was then performed on the differentially abundant metabolites (**Supplementary Table 5**). The results showed that the differential metabolites of early-stage NSCLC and HC were mainly involved 20 pathways (**Figure 7**), including sphingolipid metabolism, sphingolipid signaling pathway, primary bile acid biosynthesis, the pentose phosphate pathway, carbon metabolism, arginine biosynthesis, phenylalanine, tyrosine and tryptophan biosynthesis, etc.

### Multi-omics analysis revealed differences between HC and early-stage NSCLC groups

**Figure 8:**
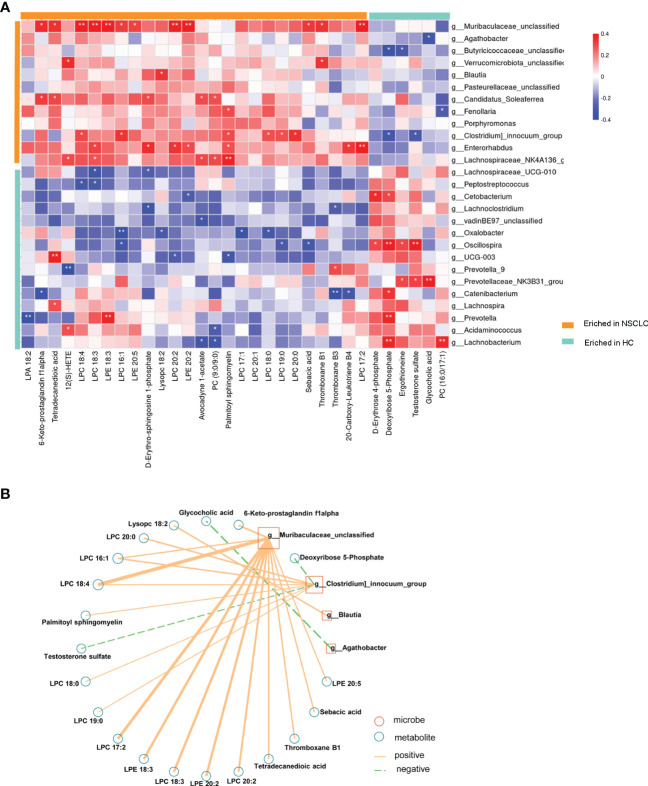
Multi-omics approaches revealed microbiota-metabolite interactions in early-stage NSCLC patients Heatmap demonstrates the correlations between 27 differentially abundant genera and 32 differentially abundant metabolites (Spearman’s correlation analysis). P-value, *p<0.05; **p<0.01; ***p<0.001 **(B)** Early-stage NSCLC associated networks based on integrated fecal microbiome and serum metabolome.

To further investigate microbiota-metabolite interactions associated with early-stage NSCLC, we assessed correlations between 27 genera and 32 metabolites (**Figure 8A**; **Supplementary Table 6**). The results showed that the abundance of several microbial genera in the early-stage NSCLC group were positively correlated with serum metabolite levels (Sperman’s correlation analysis, P<0.05, **Figure 8A**). Then, based on the above microbiome data, a co-occurrence network was constructed to elucidate the major interactions between the early-stage NSCLC associated microbiome and metabolites (**Figure 8B**). The results showed correlations between *Muribaculacead*, *Clostridium*, *Blautia*, *Agathobacter* and the related metabolites. From the graph, *Muribaculacea* and *Clostridium* seemed to be the core genera given that they were positively correlated with metabolites enriched in early-stage NSCLC and negatively correlated with certain metabolites enriched in HC (eg, Deoxyribose 5-Phosphate and Testosterone sulfate).

## Discussion

Lung cancer is the malignant tumor with the highest morbidity and mortality worldwide. NSCLC, the most common form of lung cancer, has a poor prognosis mainly because it is diagnosed at an advanced stage. One way to improve outcomes for patients with NSCLC is early diagnosis. With the development of imaging technologies such as CT imaging, positron emission tomography-computed tomography (PET-CT), and magnetic resonance imaging (MRI), the detection rate of early-stage NSCLC has increased significantly. However, no effective early-stage NSCLC biomarkers are currently available. In this study, we explored the changes in gut microbiota and serum metabolic profiles of patients with early-stage NSCLC, and combined these two omics to search for possible pathogenesis and potential biomarkers. At the same time, we also identified for the first time the characteristics of the intestinal flora of lung adenocarcinoma patients with different grades under the new grading system (Moreira et al., 2020; Deng et al., 2021), which is of great significance for precise treatment and control of prognosis.

Changes in gut flora abundance are closely related to the occurrence and development of cancer (Schwabe and Jobin, 2013; Garrett, 2015). In the present study, we provided evidence that early-stage NSCLC patients have lower abundances of *Bacteroidota* and *Actinobacteriota*, while relatively higher abundances of *Firmicutes* and *Proteobacteria* (**Figure 1B**), suggesting the potential links between gut bacteria and early-stage NSCLC. In general, dysregulation of gastrointestinal metabolism is repeatedly associated with a decreased *Firmicutes*/*Bacteroidota* ratio (Etxeberria et al., 2015; Li et al., 2018), which was found opposite in our study. We attempted to identify the reasons for the differences from the populations included and the experimental design. Since our subjects were all newly diagnosed and treated patients and their relatives represented healthy controls, the influence of genetic factors was excluded. We consider that the differences may be due to subject selection criteria or patient heterogeneity. On the other hand, previous studies included lung cancer patients with multiple pathological types (such as small cell lung cancer, NSCLC, etc.) or with different stages (such as advanced lung cancer, etc.). While in this study, we focused on exploring the gut microbiota of patients with early-stage NSCLC.

In the search for key discriminating microorganisms, it was found that *Agathobacter* and *Blautia* were the prominent differential genera of early-stage NSCLC (**Figure 4B**). Previous clinical studies have shown that two butyrate-producing gut bacteria (*Agathobacter* and *Blautia*) can favorably modulate the host immune response, were enriched in advanced NSCLC patients with better prognosis, and could become potential biomarkers for metastatic NSCLC patients treated with immune checkpoint inhibitors (Hakozaki et al., 2020; Martini et al., 2022). Consistently, *Clostridia*, another significantly genus increased in early-stage NSCLC, is thought to produce short-chain fatty acids (SCFAs) that provide essential nutrients and energy to colonic epithelial cells, induce regulatory T cells, and have anti-inflammatory effects by enhancing epithelial barrier integrity (Scaldaferri et al., 2013). All these indicated that the gut microbiota of patients with early-stage NSCLC is closely related to host immunity. Since multiple gut microbiota can disrupt host homeostasis by affecting the level of the host immune system, changes in this balance will lead to chronic inflammation and immune-related diseases, thereby promoting or attenuating the carcinogenic process (Kau et al., 2011; Zeng et al., 2016; Khan et al., 2020). In our study, the subjects were all early-stage NSCLC patients, and the abundance of these immune-related gut microbiota was significantly increased, which may slow down the further development of NSCLC by modulating the host immune system. Correspondingly, the key differential genus in HC were *Prevotella_9*, *Prevotella*, *Lachnospira* and *Catenibacterium* (**Figure 4B**). *Prevotella* belongs to the *Prevotaceae* family of the genus *Bacteroides*, which has a variety of bacterial species and is the dominant genus in the human gut. Furthermore, studies have shown that *Prevotella* decreases with lung cancer progression (Qin et al., 2022) and that changes in NSCLC patients are associated with response to immunotherapy (Jin et al., 2019b). *Lachnospira* was reported as a “favorable” gut microbiome that protects the host from cancer by producing butyrate, which plays an important role in suppressing tumor growth, regulating immunity, and participating in anti-inflammatory responses (Daniel et al., 2017). Previous studies found that both *Prevotella* and *Lachnospira* were decreased in lung cancer patients (Liu et al., 2019; Zhang et al., 2019; Qin et al., 2022), which is consistent with our findings (**Figure 3B**). These results suggested that tumor development is intricately linked to the immune system (Hanahan and Weinberg, 2011), and that carcinogenesis is often caused by dysbiosis rather than by the activity of specific pathogenic microorganisms (Matson et al., 2018; Jin et al., 2019a).

Adenocarcinoma is the most common pathological type in NSCLC. A novel grading system for lung adenocarcinoma proposed by IASLC will help identify prognostic groups and provide a common approach to prognostic stratification of lung adenocarcinoma patients who may benefit from emerging management and treatment options. This study was the first to identify specific gut microbiota in patients with different grades of invasive lung adenocarcinoma (**Figures 4C, D**), which is of great help in understanding cancer progression and prognosis in patients with lung adenocarcinoma more accurately.

Differences in the microbiome may not be used to clearly explain the role of the microbiome in health and disease (Integrative, 2014). Therefore, the use of a prospective multi-omics approach combined with comprehensive analysis of microbes and metabolites may be a way to reveal disease pathogenesis. In this study, compared with healthy people, serum glycerophospholipids (eg: LPC 20:2, LPC 18:0, LPC 18:4, LPE 20:2, LPC 20:1, LPC 16:1, LPC 20:0, LPA 18:2, etc.) were significantly higher in early-stage NSCLC patients (**Figure 6**). Glycerophospholipids are one of the main components of cell membranes, and are involved in many important life processes such as cell transmembrane transport, energy metabolism, signal transduction and cancer development (Lee et al., 2012; Santos and Schulze, 2012). High serum phospholipids and fatty acids in lung cancer patients have been previously reported (Ros-Mazurczyk et al., 2017; Zhang et al., 2020b), and our findings were consistent with previous studies (Zhao et al., 2021). Analysis of pathway enrichment using differential metabolites found that sphingolipid metabolism and sphingolipid signaling pathways were enriched in early-stage NSCLC vs HC. Sphingolipid metabolism has been shown to be the most dysregulated pathway in NSCLC patients (Petrache and Berdyshev, 2016), and alterations in gene expression patterns in this metabolic pathway were found to be strongly associated with poor prognosis in NSCLC patients (Meng et al., 2021). Sphingolipids (D-erythrosphingosine 1-phosphate and palmitoyl sphingomyelin), the metabolites significantly upregulated in early-stage NSCLC (**Figure 7**), can regulate various biological processes by controlling the signaling functions in cancer cell signaling networks, such as growth, proliferation, migration, invasion and/or metastasis (Hannun and Bell, 1987; Dressler et al., 1992). Currently, emerging therapeutic strategies targeting enzymes involved in sphingolipid metabolism and/or signaling for cancer therapy are presented. Furthermore, our results found primary bile acid biosynthesis and bile secretion pathways enriched in early-stage NSCLC vs HC (**Figure 7**). Disorders of bile acid metabolism have been shown to be associated with poor prognosis and promote the further development of aggressive lung adenocarcinoma (Nie et al., 2021). In this study, a multi-omics analysis of changes in the microbiome and metabolome was performed, and it was found that the abundance of gut microbiota was closely related to serum metabolic activity. For example, *Clostricium*, one of the genera with significant higher abundance in the early-stage NSCLC group in the LEfSe analysis (**Figure 4B**), was positively correlated with multiple glycerophospholipid (LPC 20:0, LPC 16:1, LPC 18:4, LPC 18:0, LPC 19:0). Moreover, *Muribaculaceae*, another characteristic microorganism of early-stage NSCLC (**Figure 3A**), was found to be associated with various phospholipids (LPC 18:4, LPC 17:2, LPE 20:2, LPC 20:2, etc.) and faty acyl (Sebacic acid, Tetradecanedioic acid). Previous studies found that *Muribaculaceae* is an important predictor of intestinal short-chain fatty acid concentration and that its acetate products regulate animal fat metabolism (Ormerod et al., 2016; Smith et al., 2019). These findings have potential clinical implications for patients with early-stage NSCLC.

## Conclusion

In summary, our results suggested that abnormalities in gut microbiota and metabolomics are closely related to the occurrence and development of early-stage NSCLC. Our multi-omics analysis further discovered the possible relationship between certain gut microbiota and serum phospholipids and fatty acids in early-stage NSCLC patients, and provided a basis for future research on the pathogenesis and treatment of NSCLC. It is worth mentioning that this study has the following limitations. First of all, our sample size for IPA grading is relatively small, and more sample data is needed to support it. On the other hand, most of the patients in this study were from northeastern China, which may have a certain impact on the progression of lung disease due to the colder regions and poor air quality in winter. In addition, the microbiome of this study was based on 16sRNA gene sequencing, which may be less comprehensive than metagenomic sequencing. Importantly, more later functional experiments are needed to further verify the possible targets screened in this study, so as to provide a stronger theoretical basis for the screening of early-stage NSCLC targets.

## Data availability statement

The datasets presented in this study are deposited in online repositories. The names of the repositories and accession numbers can be found below: https://data.mendeley.com/datasets/hw576fjg9s; https://data.mendeley.com/datasets/fpnmx3f5tp.

## Ethics statement

The studies involving human participants were reviewed and approved by Ethics Committee of the Third Affiliated Hospital of Harbin Medical University. The patients/participants provided their written informed consent to participate in this study.

## Author contributions

BN and SX contributed to conception and design of the study. BN, XK, and YY performed the experiment and statistical analysis. BN wrote the draft of the manuscript. SX helped revise the manuscript. BF and FZ helped perform the analysis with constructive discussions. All authors contributed to manuscript revision and approved the submitted version.

## References

[B1] Barri T. Dragsted L. O. (2013). UPLC-ESI-QTOF/MS and multivariate data analysis for blood plasma and serum metabolomics: effect of experimental artefacts and anticoagulant. Anal. Chim. Acta 768, 118–128. doi: 10.1016/j.aca.2013.01.015 23473258

[B2] Caporaso J. G. Kuczynski J. Stombaugh J. Bittinger K. Bushman F. D. Costello E. K.. (2010). QIIME allows analysis of high-throughput community sequencing data. Nat. Methods 7 (5), 335–336. doi: 10.1038/nmeth.f.303 20383131 PMC3156573

[B3] Daniel S. G. Ball C. L. Besselsen D. G. Doetschman T. Hurwitz B. L. (2017). Functional changes in the gut microbiome contribute to transforming growth factor beta-deficient colon cancer. mSystems 2 (5), e00065-17. doi: 10.1128/mSystems.00065-17 PMC5613170 28951889

[B4] Deng C. Zheng Q. Zhang Y. Jin Y. Shen X. Nie X.. (2021). Validation of the novel international association for the study of lung cancer grading system for invasive pulmonary adenocarcinoma and association with common driver mutations. J. Thorac. Oncol. 16 (10), 1684–1693. doi: 10.1016/j.jtho.2021.07.006 34302987

[B5] Dressler K. A. Mathias S. Kolesnick R. N. (1992). Tumor necrosis factor-alpha activates the sphingomyelin signal transduction pathway in a cell-free system. Science 255 (5052), 1715–1718. doi: 10.1126/science.1313189 1313189

[B6] Duma N. Santana-Davila R. Molina J. R. (2019). Non-small cell lung cancer: Epidemiology, screening, diagnosis, and treatment. Mayo. Clin. Proc. 94 (8), 1623–1640. doi: 10.1016/j.mayocp.2019.01.013 31378236

[B7] Dumas A. Bernard L. Poquet Y. Lugo-Villarino G. Neyrolles O. (2018). The role of the lung microbiota and the gut-lung axis in respiratory infectious diseases. Cell Microbiol. 20 (12), e12966. doi: 10.1111/cmi.12966 30329198

[B8] Etxeberria U. Arias N. Boque N. Macarulla M. T. Portillo M. P. Martinez J. A.. (2015). Reshaping faecal gut microbiota composition by the intake of trans-resveratrol and quercetin in high-fat sucrose diet-fed rats. J. Nutr. Biochem. 26 (6), 651–660. doi: 10.1016/j.jnutbio.2015.01.002 25762527

[B9] Garrett W. S. (2015). Cancer and the microbiota. Science 348 (6230), 80–86. doi: 10.1126/science.aaa4972 25838377 PMC5535753

[B10] Hakozaki T. Richard C. Elkrief A. Hosomi Y. Benlaifaoui M. Mimpen I.. (2020). The gut microbiome associates with immune checkpoint inhibition outcomes in patients with advanced non-small cell lung cancer. Cancer Immunol. Res. 8 (10), 1243–1250. doi: 10.1158/2326-6066.CIR-20-0196 32847937

[B11] Hanahan D. Weinberg R. A. (2011). Hallmarks of cancer: the next generation. Cell 144 (5), 646–674. doi: 10.1016/j.cell.2011.02.013 21376230

[B12] Hannun Y. A. Bell R. M. (1987). Lysosphingolipids inhibit protein kinase c: implications for the sphingolipidoses. Science 235 (4789), 670–674. doi: 10.1126/science.3101176 3101176

[B13] Herbst R. S. Morgensztern D. Boshoff C. (2018). The biology and management of non-small cell lung cancer. Nature 553 (7689), 446–454. doi: 10.1038/nature25183 29364287

[B14] Hou L. Wang T. Chen D. She Y. Deng J. Yang M.. (2022). Prognostic and predictive value of the newly proposed grading system of invasive pulmonary adenocarcinoma in Chinese patients: a retrospective multicohort study. Mod. Pathol. 35 (6), 749–756. doi: 10.1038/s41379-021-00994-5 35013526

[B15] Integrative H.M.P.R.N.C. (2014). The integrative human microbiome project: dynamic analysis of microbiome-host omics profiles during periods of human health and disease. Cell Host Microbe 16 (3), 276–289. doi: 10.1016/j.chom.2014.08.014 25211071 PMC5109542

[B17] Jin Y. Dong H. Xia L. Yang Y. Zhu Y. Shen Y.. (2019b). The diversity of gut microbiome is associated with favorable responses to anti-programmed death 1 immunotherapy in Chinese patients with NSCLC. J. Thorac. Oncol. 14 (8), 1378–1389. doi: 10.1016/j.jtho.2019.04.007 31026576

[B16] Jin C. Lagoudas G. K. Zhao C. Bullman S. Bhutkar A. Hu B.. (2019a). Commensal microbiota promote lung cancer development *via* gammadelta T cells. Cell 176 (5), 998–1013 e1016. doi: 10.1016/j.cell.2018.12.040 30712876 PMC6691977

[B18] Kau A. L. Ahern P. P. Griffin N. W. Goodman A. L. Gordon J. I. (2011). Human nutrition, the gut microbiome and the immune system. Nature 474 (7351), 327–336. doi: 10.1038/nature10213 21677749 PMC3298082

[B19] Keely S. Talley N. J. Hansbro P. M. (2012). Pulmonary-intestinal cross-talk in mucosal inflammatory disease. Mucosal Immunol. 5 (1), 7–18. doi: 10.1038/mi.2011.55 22089028 PMC3243663

[B20] Khan M. A. W. Ologun G. Arora R. McQuade J. L. Wargo J. A. (2020). Gut microbiome modulates response to cancer immunotherapy. Dig. Dis. Sci. 65 (3), 885–896. doi: 10.1007/s10620-020-06111-x 32067144 PMC7678709

[B21] Lee G. K. Lee H. S. Park Y. S. Lee J. H. Lee S. C. Lee J. H.. (2012). Lipid MALDI profile classifies non-small cell lung cancers according to the histologic type. Lung Cancer 76 (2), 197–203. doi: 10.1016/j.lungcan.2011.10.016 22099218

[B22] Li Y. Liu T. Yan C. Xie R. Guo Z. Wang S.. (2018). Diammonium glycyrrhizinate protects against nonalcoholic fatty liver disease in mice through modulation of gut microbiota and restoration of intestinal barrier. Mol. Pharm. 15 (9), 3860–3870. doi: 10.1021/acs.molpharmaceut.8b00347 30036479

[B23] Liu F. Li J. Guan Y. Lou Y. Chen H. Xu M.. (2019). Dysbiosis of the gut microbiome is associated with tumor biomarkers in lung cancer. Int. J. Biol. Sci. 15 (11), 2381–2392. doi: 10.7150/ijbs.35980 31595156 PMC6775324

[B24] Lu H. Gao N. L. Tong F. Wang J. Li H. Zhang R.. (2021). Alterations of the human lung and gut microbiomes in non-small cell lung carcinomas and distant metastasis. Microbiol. Spectr. 9 (3), e0080221. doi: 10.1128/Spectrum.00802-21 34787462 PMC8597645

[B25] Martini G. Ciardiello D. Dallio M. Famiglietti V. Esposito L. Corte C. M. D.. (2022). Gut microbiota correlates with antitumor activity in patients with mCRC and NSCLC treated with cetuximab plus avelumab. Int. J. Cancer 151 (3), 473–480. doi: 10.1002/ijc.34033 35429341 PMC9321613

[B26] Matson V. Fessler J. Bao R. Chongsuwat T. Zha Y. Alegre M. L.. (2018). The commensal microbiome is associated with anti-PD-1 efficacy in metastatic melanoma patients. Science 359 (6371), 104–108. doi: 10.1126/science.aao3290 29302014 PMC6707353

[B27] Meng Q. Hu X. Zhao X. Kong X. Meng Y. M. Chen Y.. (2021). A circular network of coregulated sphingolipids dictates lung cancer growth and progression. EBioMedicine 66, 103301. doi: 10.1016/j.ebiom.2021.103301 33813137 PMC8047482

[B28] Moreira A. L. Ocampo P. S. S. Xia Y. Zhong H. Russell P. A. Minami Y.. (2020). A grading system for invasive pulmonary adenocarcinoma: A proposal from the international association for the study of lung cancer pathology committee. J. Thorac. Oncol. 15 (10), 1599–1610. doi: 10.1016/j.jtho.2020.06.001 32562873 PMC8362286

[B29] Nie M. Yao K. Zhu X. Chen N. Xiao N. Wang Y.. (2021). Evolutionary metabolic landscape from preneoplasia to invasive lung adenocarcinoma. Nat. Commun. 12 (1), 6479. doi: 10.1038/s41467-021-26685-y 34759281 PMC8580984

[B30] Ormerod K. L. Wood D. L. Lachner N. Gellatly S. L. Daly J. N. Parsons J. D.. (2016). Genomic characterization of the uncultured bacteroidales family S24-7 inhabiting the guts of homeothermic animals. Microbiome 4 (1), 36. doi: 10.1186/s40168-016-0181-2 27388460 PMC4936053

[B31] Petrache I. Berdyshev E. V. (2016). Ceramide signaling and metabolism in pathophysiological states of the lung. Annu. Rev. Physiol. 78, 463–480. doi: 10.1146/annurev-physiol-021115-105221 26667073 PMC13200783

[B33] Qin X. Bi L. Yang W. He Y. Gu Y. Yang Y.. (2022). Dysbiosis of the gut microbiome is associated with histopathology of lung cancer. Front. Microbiol. 13. doi: 10.3389/fmicb.2022.918823 PMC9237568 35774470

[B32] Qin J. Li R. Raes J. Arumugam M. Burgdorf K. S. Manichanh C.. (2010). A human gut microbial gene catalogue established by metagenomic sequencing. Nature 464 (7285), 59–65. doi: 10.1038/nature08821 20203603 PMC3779803

[B34] Reyon D. Tsai S. Q. Khayter C. Foden J. A. Sander J. D. Joung J. K. (2012). FLASH assembly of TALENs for high-throughput genome editing. Nat. Biotechnol. 30 (5), 460–465. doi: 10.1038/nbt.2170 22484455 PMC3558947

[B35] Ros-Mazurczyk M. Jelonek K. Marczyk M. Binczyk F. Pietrowska M. Polanska J.. (2017). Serum lipid profile discriminates patients with early lung cancer from healthy controls. Lung Cancer 112, 69–74. doi: 10.1016/j.lungcan.2017.07.036 29191603

[B36] Santoni M. Piva F. Conti A. Santoni A. Cimadamore A. Scarpelli M.. (2018). Re: Gut microbiome influences efficacy of PD-1-based immunotherapy against epithelial tumors. Eur. Urol. 74 (4), 521–522. doi: 10.1016/j.eururo.2018.05.033 29891391

[B37] Santos C. R. Schulze A. (2012). Lipid metabolism in cancer. FEBS J. 279 (15), 2610–2623. doi: 10.1111/j.1742-4658.2012.08644.x 22621751

[B38] Scaldaferri F. Gerardi V. Lopetuso L. R. Del Zompo F. Mangiola F. Boskoski I.. (2013). Gut microbial flora, prebiotics, and probiotics in IBD: their current usage and utility. BioMed. Res. Int. 2013, 435268. doi: 10.1155/2013/435268 23991417 PMC3749555

[B39] Schwabe R. F. Jobin C. (2013). The microbiome and cancer. Nat. Rev. Cancer 13 (11), 800–812. doi: 10.1038/nrc3610 24132111 PMC3986062

[B40] Segata N. Izard J. Waldron L. Gevers D. Miropolsky L. Garrett W. S.. (2011). Metagenomic biomarker discovery and explanation. Genome Biol. 12 (6), R60. doi: 10.1186/gb-2011-12-6-r60 21702898 PMC3218848

[B41] Siegel R. L. Miller K. D. Jemal A. (2018). Cancer statistics 2018. CA Cancer J. Clin. 68 (1), 7–30. doi: 10.3322/caac.21442 29313949

[B42] Smith B. J. Miller R. A. Ericsson A. C. Harrison D. C. Strong R. Schmidt T. M. (2019). Changes in the gut microbiome and fermentation products concurrent with enhanced longevity in acarbose-treated mice. BMC Microbiol. 19 (1), 130. doi: 10.1186/s12866-019-1494-7 31195972 PMC6567620

[B43] Travis W. D. Brambilla E. Nicholson A. G. Yatabe Y. Austin J. H. M. Beasley M. B.. (2015). The 2015 world health organization classification of lung tumors: Impact of genetic, clinical and radiologic advances since the 2004 classification. J. Thorac. Oncol. 10 (9), 1243–1260. doi: 10.1097/JTO.0000000000000630 26291008

[B44] Want E. J. O'Maille G. Smith C. A. Brandon T. R. Uritboonthai W. Qin C.. (2006). Solvent-dependent metabolite distribution, clustering, and protein extraction for serum profiling with mass spectrometry. Anal. Chem. 78 (3), 743–752. doi: 10.1021/ac051312t 16448047

[B45] Wen B. Mei Z. Zeng C. Liu S. (2017). metaX: a flexible and comprehensive software for processing metabolomics data. BMC Bioinf. 18 (1), 183. doi: 10.1186/s12859-017-1579-y PMC5361702 28327092

[B46] Wikoff W. R. Anfora A. T. Liu J. Schultz P. G. Lesley S. A. Peters E. C.. (2009). Metabolomics analysis reveals large effects of gut microflora on mammalian blood metabolites. Proc. Natl. Acad. Sci. U.S.A. 106 (10), 3698–3703. doi: 10.1073/pnas.0812874106 19234110 PMC2656143

[B47] Wilmanski T. Rappaport N. Earls J. C. Magis A. T. Manor O. Lovejoy J.. (2019). Blood metabolome predicts gut microbiome alpha-diversity in humans. Nat. Biotechnol. 37 (10), 1217–1228. doi: 10.1038/s41587-019-0233-9 31477923

[B48] Zeng M. Y. Cisalpino D. Varadarajan S. Hellman J. Warren H. S. Cascalho M.. (2016). Gut microbiota-induced immunoglobulin G controls systemic infection by symbiotic bacteria and pathogens. Immunity 44 (3), 647–658. doi: 10.1016/j.immuni.2016.02.006 26944199 PMC4794373

[B49] Zhang D. Li S. Wang N. Tan H. Y. Zhang Z. Feng Y. (2020a). The cross-talk between gut microbiota and lungs in common lung diseases. Front. Microbiol. 11. doi: 10.3389/fmicb.2020.00301 PMC7052046 32158441

[B51] Zhang W. Luo J. Dong X. Zhao S. Hao Y. Peng C.. (2019). Salivary microbial dysbiosis is associated with systemic inflammatory markers and predicted oral metabolites in non-small cell lung cancer patients. J. Cancer 10 (7), 1651–1662. doi: 10.7150/jca.28077 31205521 PMC6548009

[B50] Zhang L. Zheng J. Ahmed R. Huang G. Reid J. Mandal R.. (2020b). A high-performing plasma metabolite panel for early-stage lung cancer detection. Cancers (Basel). 12 (3), 622. doi: 10.3390/cancers12030622 PMC7139410 32156060

[B52] Zhao F. An R. Wang L. Shan J. Wang X. (2021). Specific gut microbiome and serum metabolome changes in lung cancer patients. Front. Cell Infect. Microbiol. 11. doi: 10.3389/fcimb.2021.725284 PMC8435782 34527604

[B53] Zheng Y. Fang Z. Xue Y. Zhang J. Zhu J. Gao R.. (2020). Specific gut microbiome signature predicts the early-stage lung cancer. Gut. Microbes 11 (4), 1030–1042. doi: 10.1080/19490976.2020.1737487 32240032 PMC7524275

[B54] Zhuang H. Cheng L. Wang Y. Zhang Y. K. Zhao M. F. Liang G. D.. (2019). Dysbiosis of the gut microbiome in lung cancer. Front. Cell Infect. Microbiol. 9. doi: 10.3389/fcimb.2019.00112 PMC6489541 31065547

