# Supplementary material for: Combined analysis of gut microbiome and serum metabolomics reveals novel biomarkers in patients with early-stage non-small cell lung cancer
Source: Front Cell Infect Microbiol. 2023 Jan 20;13:1091825. doi: 10.3389/fcimb.2023.1091825 (PMC9895385; doi:10.3389/fcimb.2023.1091825)
Supplement: Supplementary file 1 [file DataSheet_1.zip › Data sheet/Supplementary_Material.docx]

Supplementary Material

# Supplementary Tables

Supplementary Table 1. ASV signature sequence of each sample

Supplementary Table 2. The gut microbiota proportions in the HC and early-stage NSCLC group at the phylum, species, and genus levels

Supplementary Table 3. LC-MS-based metabolite identified in HC and early-stage NSCLC group

Supplementary Table 4. The significantly different metabolites identified between the HC and early-stage NSCLC groups

Supplementary Table 5. The KEGG pathway enrichment analysis between the HC and early-stage NSCLC groups

Supplementary Table 6. Spearman correlation (r) values for differential metabolites and differential microbes

# Supplementary Figures

#
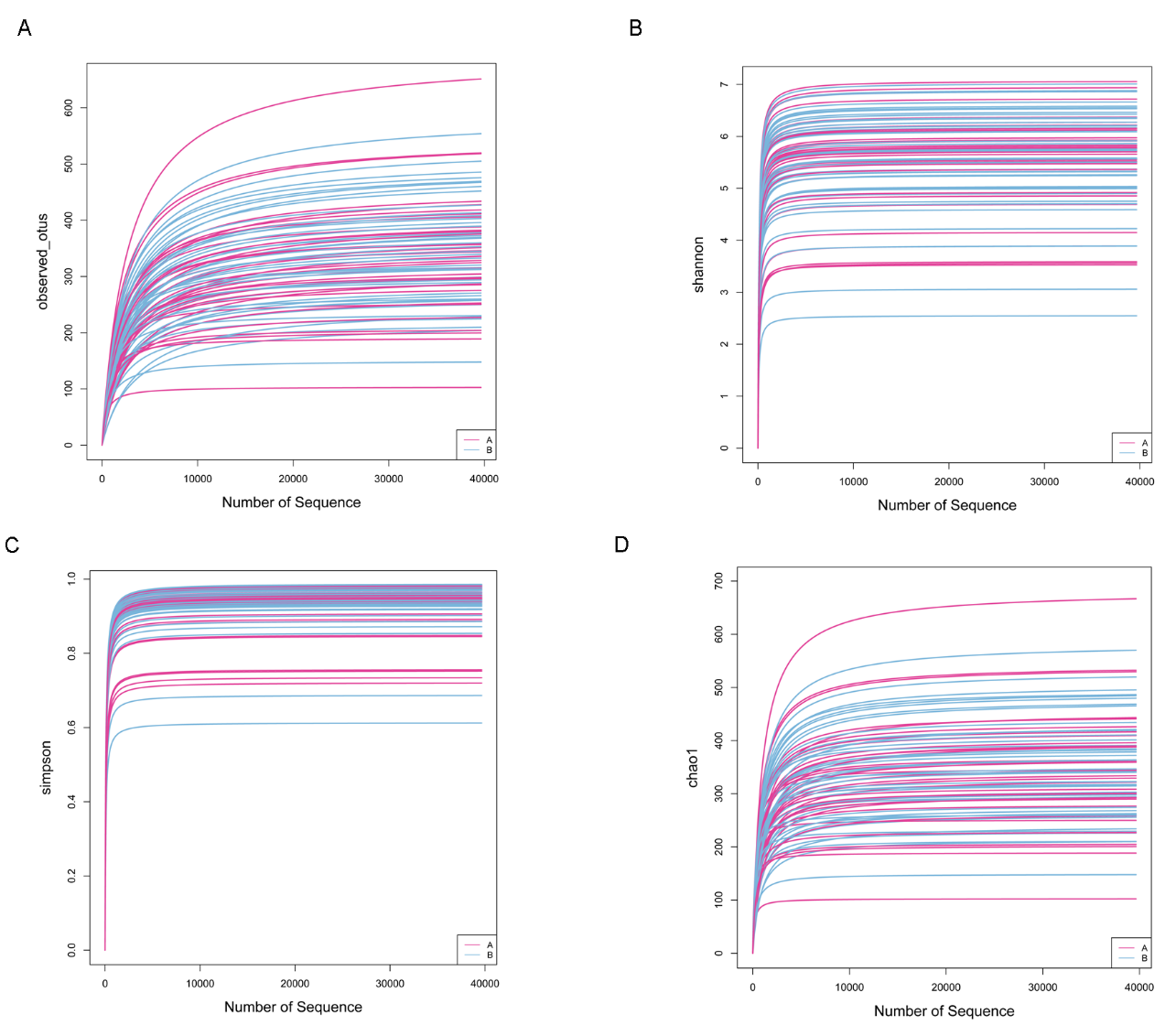


**Supplementary Figure 1.** Rarefaction curve evaluating the relative bacterial richness

Observed_otus, Shannon, and Simpson's indices and Chao 1 were used to assess the alpha diversity of gut microbiota between early-stage NSCLC patients and HC. Each curve represents a sample and is marked in blue and purple, respectively (A for HC, B for early-stage NSCLC).


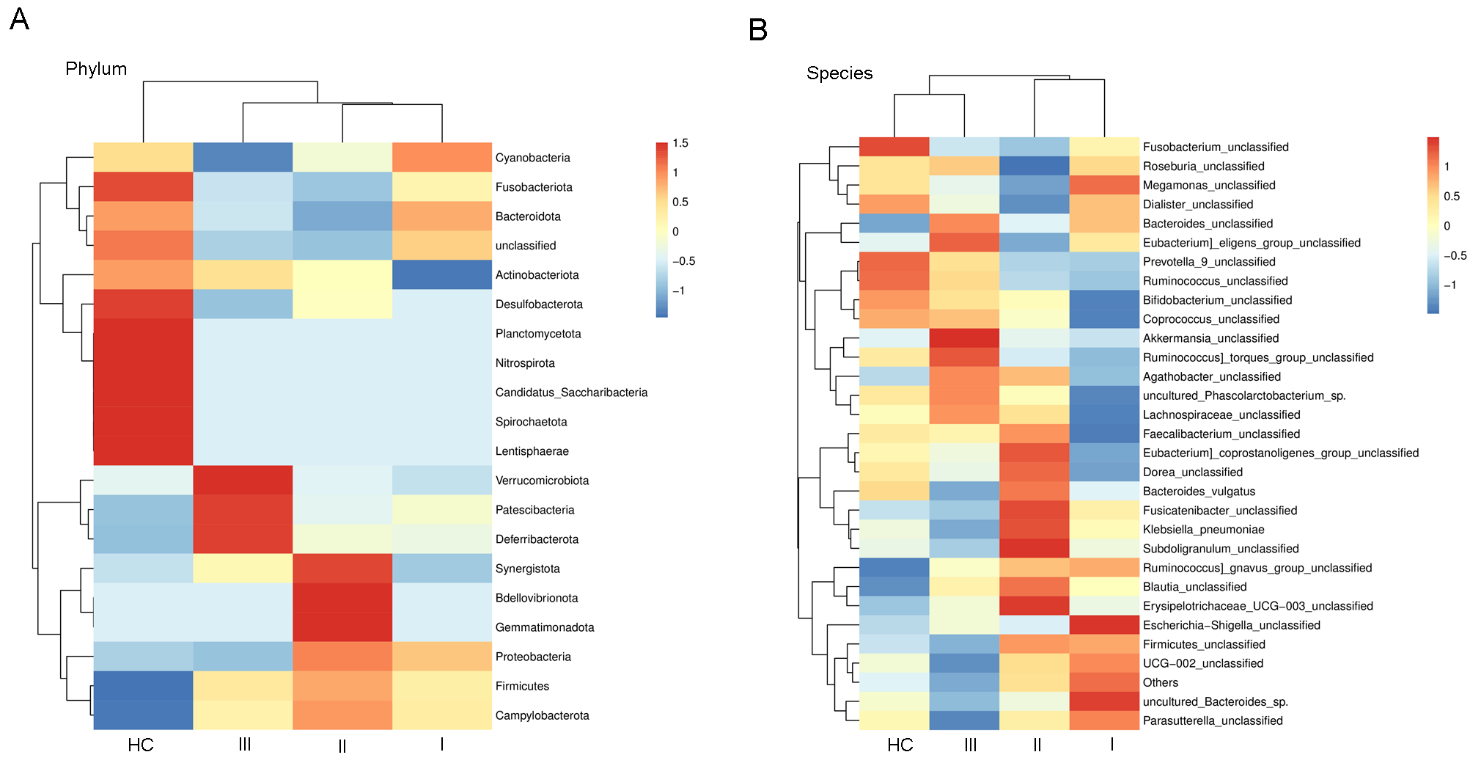


**Supplementary Figure 2.** Differences in gut microbiota abundance across different IPA grades and HC

(A)Heatmap showing the relative abundance of differential microorganisms at the phylum level across the four groups of samples. (B) Heatmap showing the relative abundance of differential microorganisms across the four groups at the species level.


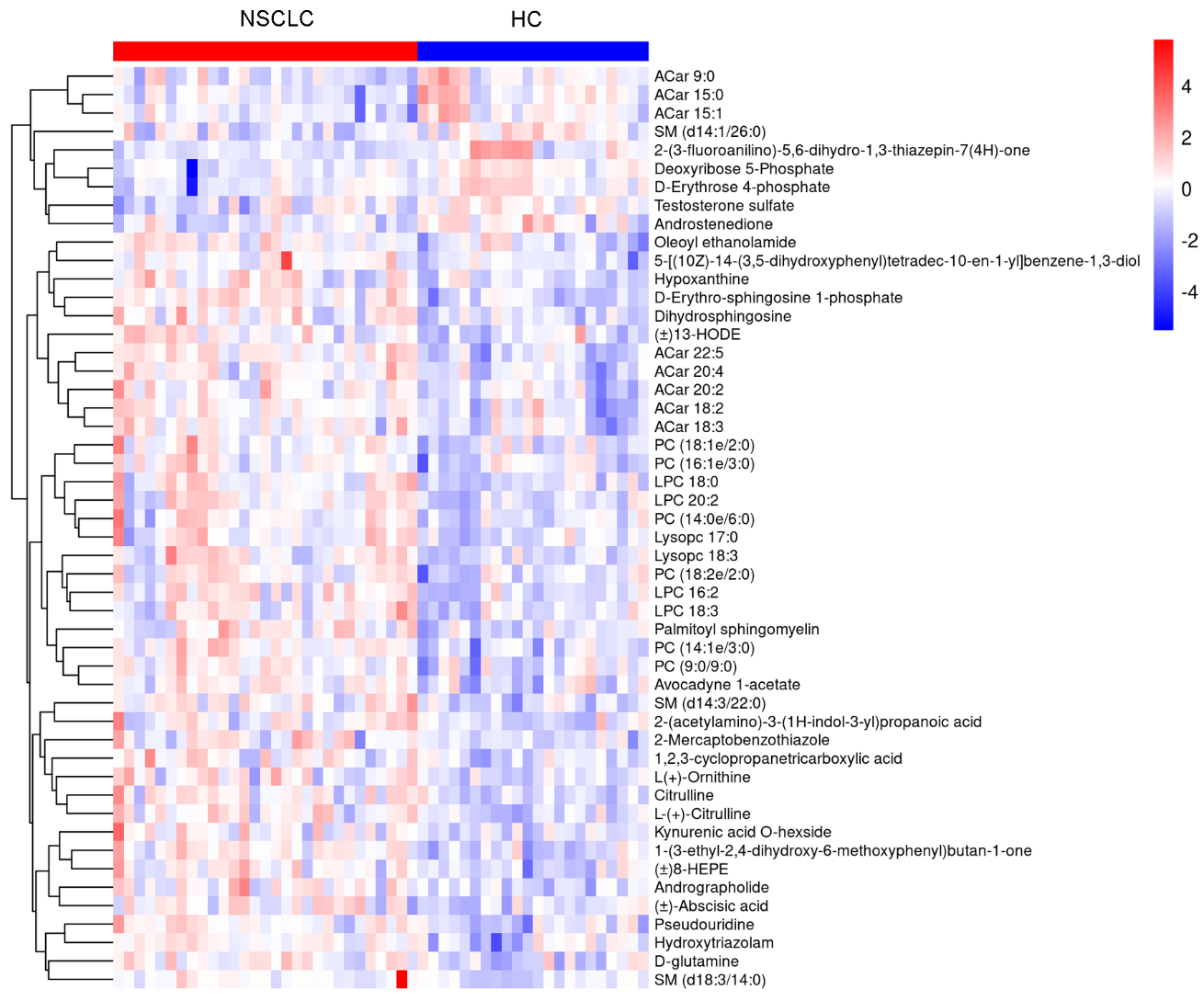


**Supplementary Figure 3.** The heat map of top 50 differentially abundant metabolites based on the relative abundance (P<0.05).
